# Arguments for Using Direct Oral Anticoagulants in Cancer-Related Venous Thromboembolism

**DOI:** 10.3390/healthcare9101287

**Published:** 2021-09-28

**Authors:** Roxana Mihaela Chiorescu, Mihaela Mocan, Mirela Anca Stoia, Anamaria Barta, Cerasela Mihaela Goidescu, Stefan Chiorescu, Anca Daniela Farcaş

**Affiliations:** 1 Internal Medicine Department, Iuliu Hatieganu University of Medicine and Pharmacy, 400012 Cluj-Napoca, Romania; chiorescu.roxana@umfcluj.ro (R.M.C.); mirelastoia@yahoo.com (M.A.S.); apicrek@yahoo.com (A.B.); ceraselagoidescusava@yahoo.com (C.M.G.); ancafarcas@yahoo.com (A.D.F.); 2 Department of Internal Medicine, Emergency Clinical County Hospital, 400006 Cluj-Napoca, Romania; 3 Department of Cardiology, Emergency Clinical County Hospital, 400006 Cluj-Napoca, Romania; 4 Department of Cardiology, “Nicolae Stăncioiu” Heart Institute, 400001 Cluj-Napoca, Romania; 5 Department of Cardiology, Military Emergency Hospital “C. Papilian”, 400132 Cluj-Napoca, Romania; 6 Surgery Department, Iuliu Hatieganu University of Medicine and Pharmacy, 400012 Cluj-Napoca, Romania; stefanc74@yahoo.com

**Keywords:** direct oral anticoagulants, cancer-associated venous thromboembolism

## Abstract

(1) Background: Patients with cancer with a hypercoagulable state present an increased incidence of venous thromboembolism (VTE). Neoplastic patients with concurrent VTE undergoing anticoagulant treatment face a series of issues. (2) The aim of the present paper is to systematically summarize current VTE management in patients with neoplasia and to review the current clinical evidence from meta-analyses of randomized controlled trials and guidelines regarding the administration of direct oral anticoagulants (DOACs) for cancer-associated VTE. (3) Search Strategy: We performed a review on meta-analyses of randomized controlled trials and guidelines in favor of the administration of DOACs in patients with cancer-associated VTE published in the last 6 years in the Medline (PubMed) and Embase databases. (4) Results: 21 meta-analyses, 14 randomized controlled studies comparing DOACs to VKAs and LMWH, and 7 national and international guidelines were identified. We identified five studies that show the antineoplastic effect of DOAC on experimental models. (5) Conclusions: DOACs can be seen as the first choice for VTE treatment in neoplastic patients who have a low risk of bleeding, who do not have severe renal impairment, and who are not undergoing treatments that could interact with DOAC’s mechanism of action.

## 1. Introduction

Venous thromboembolism (VTE) is the second most frequent cause of death in patients with neoplasia undergoing chemotherapy, while the main cause of death in these patients is the progression of the neoplasm [1].

The association of cancer with VTE determines a 30 times higher risk of death as compared to the general population. The association of cancer with VTE increases the rate of mortality exponentially, which is 10 times higher than in the case of VTE patients without neoplasia and four times higher than in neoplastic patients without VTE [2].

The hypercoagulable state in neoplasia can be explained by the multiple physio-pathological mechanisms present in oncogenesis: (1) the production of procoagulant and angiogenic substances induced by tumor hypoxemia; (2) the changes in the thrombocyte–endothelium interaction and in the cascade of coagulation and fibrinolysis, i.e., there is an increase in the concentration of thrombocyte-activating factors (P-selectin, soluble CD40 ligand, and platelet factor); (3) changes in the concentration of the tissue factor responsible for the initiation of coagulation; (4) the synthesis of a protease capable of directly activating factor Xa [3].

Anticoagulant treatment in patients with cancer-associated VTE faces a number of problems such as increased risk of bleeding, high risk of VTE recurrence, interactions with the specific treatment of neoplasia, and the need for a long-term administration of the treatment. Recently, the standard of care of cancer-associated VTE consisted of subcutaneous low-molecular-weight heparin (LMWH) for an initial duration of 6 months, which was extended with either LMWH or vitamin K antagonists (VKA) for an indefinite duration, i.e., until the cancer was considered to be in remission [4]. The administration of LMWHs faces a series of drawbacks connected especially with the manner of administration, which leads to a decrease in treatment compliance and an increase in the risk of VTE recurrence [5].

The aim of the present paper was to systematically summarize current VTE management in patients with neoplasia and to review the current clinical evidence from meta-analyses of randomized controlled trials and guidelines in favor of the administration of DOACs in this category of patients. 

## 2. Materials and Methods

### 2.1. Search Strategy

**Figure 1:**
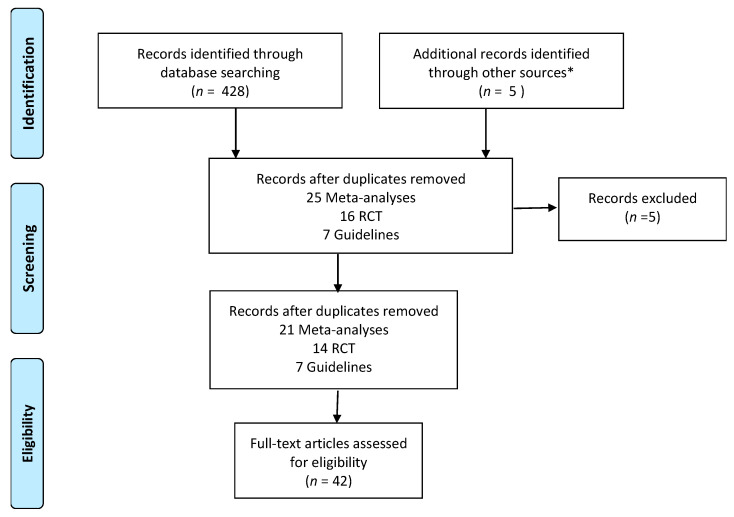
The algorithm showing the identification of articles included in the review. * Other sources: references from meta-analyses and guidelines.

The search strategy was performed according to a predefined protocol and complied with the PRISMA guidelines [6], as shown in Figure 1.

### 2.2. Information Sources

Two authors systematically searched the PubMed and Embase databases using the following combination of keywords: “cancer related thromboembolism DOAC”, “cancer thromboembolism DOAC”, “cancer thromboembolism DOAC”, “cancer thromboembolism dabigatran”, “cancer thromboembolism direct thrombin inhibitor”, “cancer thromboembolism rivaroxaban”, “cancer thromboembolism apixaban”, “cancer thromboembolism edoxaban”, “cancer thromboembolism factor Xa inhibitor”. Apart from these keywords, “the antineoplastic effect of DOAC on experimental models” was searched on Pub Med. 

### 2.3. Eligibility Criteria

We included meta-analyses of large studies and of randomized controlled trials and practice guidelines of national and international societies, referring to DOAC prescription in patients with VTE associated with neoplasia. Two authors independently evaluated the studies for possible inclusion. Non-relevant studies were excluded based on their titles and abstracts. For potentially relevant studies, a full text was obtained. The research was restricted to meta-analyses of large studies and of randomized controlled trials and practice guidelines published in the past 6 years (January 2015–December 2020).

## 3. Results

The flow diagram of the evaluation process is shown in Figure 1. The literature search yielded a total of 393 related articles. After duplicates were removed, 42 articles remained.

### 3.1. Meta-Analyses 

The 21 meta-analyses published in the past 6 years are shown in Table 1. Several meta-analyses focusing on the efficacity and safety of DOACs in cancer-associated VTE were identified. Efficiency was assessed according to the recurrence rate and safety as related to major bleeding and clinically relevant nonmajor bleeding (CRNMB). Comparisons between DOACs, LMWH, and VKAs were analyzed. The most important conclusion drawn from these extensive analyses was that DOACs demonstrate a similar efficiency and safety as compared to LMWH and VKAs in cancer-associated VTE [7,8].

### 3.2. Randomized Controlled Studies 

Randomized controlled studies comparing DOACs to VKAs and LMWH respectively offer encouraging arguments with regard to the administration of DOACs in paraneoplastic VTE patients (Table 2). Multiple clinical trials compared the efficacy and safety of DOAC vs. LMWH administration in neoplastic patients with VTE. The VTE-recurrence risk was assessed, as was the risk of fatal and nonfatal hemorrhage recurrence under the two types of anticoagulants.

### 3.3. Guidelines Published in the Past 6 Years 

Guidelines published in the past 6 years that recommend the use of DOACs in neoplastic patients with VTE are summarized in Table 3. Practice guidelines play a crucial role in modern medicine. The information found in the guidelines was extracted from RCT and meta-analyses for the clinicians to apply in real-life patients.

### 3.4. DOACs beyond Anticoagulation: A Potential Antineoplastic Effect

The studies that assessed the antineoplastic effect in experimental models are presented in Table 4. 

## 4. Discussion

### 4.1. Meta-Analysis

Some of the most recently published meta-analyses comprising thousands of patients diagnosed with cancer-associated VTE showed that those treated with DOAC had a higher risk of bleeding [21,23,25]. Most of these bleedings were minor and were found especially in patients with gastrointestinal tumors. Desai R. et al. showed that DOACs are superior to VKA or LMWH for secondary prevention of VTE in patients with cancer, although they have an increased risk for nonmajor bleeding as compared to LMWH [23]. 

The interpretation of bleeding risks associated with DOACs is complex. Bleeding events associated with DOACs seem to be mostly related to mucosal bleeding. Upper digestive cancer and the use of edoxaban and rivaroxaban were frequently associated with more bleeding events [24,26].

Moreover, bleeding with DOACs may be related to their physiological and pharmacological mechanism of action. Thus, dabigatran, rivaroxaban, and edoxaban may have a topical anticoagulant effect that favors mucosal bleeding, tartaric acid found in dabigatran has a direct caustic effect, while rivaroxaban and edoxaban have pharmacodynamic differences leading to higher peak concentration [49]. To date, DOACs have not been evaluated for the bleeding risk of each representant. However, in one previous meta-analysis (published in 2013) of 43 trials utilizing DOACs for any indication including VTE (but excluding cancer), dabigatran and rivaroxaban were found to be associated with more digestive bleeding and nonmajor bleeding as compared to apixaban [50].

In this respect, Angelini et al. showed that, in presence of neoplasia, patients treated with anticoagulation experienced more bleeding events regardless of the type of DOAC used. Other factors influencing the risk of bleeding in cancer patients were as follows: renal impairment, obesity with a BMI ≥ 40 kg/mp, the presence of metastatic disease, a moderate to severe thrombocytopenia, and tumor type. In this respect, the authors found that regardless of the type of anticoagulant, patients with primary digestive neoplasia had more bleeding events than those with nondigestive cancers [51]. 

The arguments regarding the safety of different anticoagulants in cancer patients should be established by performing a subgroup meta-analysis of bleeding risk, stratified by cancer type and DOAC type. 

### 4.2. Randomized Controlled Trials

The efficacy and safety of LMWHs as compared to VKAs was shown in the following studies: Meyer, Clot, Hull, Deitcher, and Catch [52]. In these studies, the patients treated with LMWHs presented a lower VTE recurrence rate, without an increased risk of major bleeding, as compared to those treated with VKAs [53]. However, LMWH therapy is a burden both financially and socially, requiring daily subcutaneous injections that affect the quality of life. VKAs are sometimes used in cancer patients with increased bleeding risk or VTE recurrence because of the narrow therapeutic range and possible drug interactions found in cancer patients [54].

A number of recent studies show that DOACs have the same efficacy as VKAs and exhibit a better safety profile; thus, they are a potential therapeutic option in patients with paraneoplastic VTE [29,33,55,56,57]. The most important clinical trials that compared DOACs to VKAs in patients with neoplasia are as follows: RE-COVER I and II (dabigatran with VKA), EINSTEIN PE and DVT (rivaroxaban with VKA), AMPLIFY (apixaban with VKA), and Hokusai-VTE (edoxaban with VKA). The results of these clinical trials offer encouraging arguments with regard to the administration of DOACs in paraneoplastic VTE patients (Table 2).

The SELECT-D study included 406 patients with neoplasia and VTE. The subjects were randomly divided into two groups: the first group received rivaroxaban treatment (15 mg twice daily for 3 weeks and subsequently 20 mg once daily for 6 months); the second group received dalteparin treatment (200 IU/kg for 1 month and subsequently 150 IU/kg for 2–6 months). VTE recurrence 6 months into the treatment was 11% in the group undergoing dalteparin treatment and 4% in the group treated with rivaroxaban. The cumulative risk of major hemorrhages 6 months into the treatment was 4% with dalteparin treatment as compared to 6% with rivaroxaban treatment [31].

The ADAM-VTE study included 300 patients who were randomly divided into two groups: the first group was treated with apixaban (10 mg twice daily for 7 days and subsequently 5 mg twice daily for 6 months); the second group was treated with dalteparin (200 IU/kg for 1 month and subsequently 150 IU/kg for 6 months). The results of this study showed a lower hemorrhage and VTE-recurrence risk in the group treated with apixaban (recurrent VTE was 3.4% in the group treated with apixaban as compared to 14.1% in the group treated with dalteparin, major hemorrhages were 0% in the group treated with apixaban as compared to 2.1% in the group treated with dalteparin, and the incidence rate of major and nonmajor hemorrhages was the same in both groups, i.e., 9%) [34].

The CARAVAGGIO study is characterized as a multinational, randomized, investigator-initiated, open-label, non-inferiority trial with blinded central outcome adjudication. In this study, consecutive patients with cancer diagnosed with symptomatic or incidental acute proximal VTE/PE received either oral apixaban (a dose of 10 mg twice daily for the first week, followed by 5 mg twice daily for 6 months) or subcutaneous dalteparin (a dose of 200 IU per kilogram of body weight once daily for the first month, followed by 150 IU per kilogram once daily for 6 months). The primary outcome was recurrent VTE, which was objectively confirmed. Recurrent VTE frequency was higher in the dalteparin group (7.9%) as compared to the apixaban group (5.6%). On the other hand, no statistically significant differences regarding major bleeding were identified between the two groups (3.8% in the apixaban group and 4% in the dalteparin group). Thus, apixaban proved to be both effective and safe for the treatment of CAT VTE [35]. 

The Hokusai-VTE Cancer study included 1046 patients who were randomly divided into two groups: the first group was treated with LMWHs for 5 days and subsequently with edoxaban (60 mg daily); the second group was treated with dalteparin (200 IU/kg during the first month, followed by 150 IU/kg). The edoxaban doses were adjusted to renal function and body weight (30 mg in the case of patients with an eGFR of 30–50 mL/min, weight < 60 kg, or in the case of an association with antiplatelet aggregation therapy). The risk of recurrent VTE was higher in the group treated with dalteparin, and the risk of major hemorrhages was greater in the group treated with edoxaban. The risk of recurrent VTE was 7.9% in the group treated with edoxaban as compared to 11.3% in the group under dalteparin treatment, and the risk of major hemorrhages was 6.9% in the group treated with edoxaban as compared to 4% in the group treated with dalteparin. The majority of hemorrhages in the group treated with edoxaban were gastrointestinal hemorrhages due to the gastrointestinal mucous membrane being affected by the toxic effects of the chemotherapeutic agents [30]. 

There are additional ongoing clinical studies that compare the efficacy and safety of DOAC vs. LMWH administration in neoplastic patients with VTE, e.g., CANVAS: dabigatran, rivaroxaban, apixaban, edoxaban vs. LMWH [36]; CONKO-011: rivaroxaban vs. LMWH [37]; CASTA-DIVA: rivaroxaban vs. LMWH [39]; COSIMO: rivaroxaban vs. LMWH [39].

There are several ongoing studies (one of them being the AVERT study) investigating the risk/benefit ratio in VTE prophylaxis in neoplastic patients with an intermediate and high risk of thrombosis.

### 4.3. Guidelines

In our review, we identified seven national and international guidelines that mention the baseline anticoagulant therapy in cancer patients suffering from VTE. 

Until recently, higher adherence to the treatment has been considered one of the advantages of DOACs as compared to LWMH. Patients following an anticoagulant treatment with LMWH, which are administered by subcutaneous injection twice a day, were supposed to give up their treatment more frequently in favor of VTE recurrence [46,58,59]. Early this year, Schaefer et al. showed a similarly high rate (95%) of LMWH and DOAC adherence for patients with CAT. Thus, the authors recommend that anticoagulant therapy should not be guided by the probability of treatment compliance [5].

Edoxaban, rivaroxaban, and apixaban are the only DOACs that have been compared to LMWHs in clinical studies and are accepted for cancer-associated VTE. The use of rivaroxaban in the treatment of these patients is supported by an abundance of clinical evidence. DOACs are associated with a statistically significant lower risk of VTE recurrence in neoplastic patients but with a higher bleeding risk [20,60,61]

Some of the guidelines still recommend LMWH as the first-line therapy of cancer-associated VTE, while others recommend DOACs but in appropriate situations, i.e., no drug interactions with current cancer medication, a low risk of bleeding, and normal renal function. Thus, ESC [42] and ASCO [44] suggest the use of LMWH in patients with acute VTE diagnosis (during the first 7–10 days to 6 months) but prefer rivaroxaban to LMWH in patients without gastrointestinal cancer. On the other hand, ISTH [41] and ACCP [43] suggest DOACs in selected categories of patients with acute VTE, a low bleeding risk, and no drug interaction, and suggest LMWH in patients with a high bleeding risk (digestive cancer, genitourinary cancer, and digestive mucosal abnormalities, such as esophagitis, gastritis, colitis, etc.). 

### 4.4. DOACs beyond Anticoagulation: A Potential Antineoplastic Effect?

To enhance the indication of DOACs in patients with VTE and cancer, several experimental studies were conducted with the aim of elucidating the possible antineoplastic effect of these drugs.

**Figure 2:**
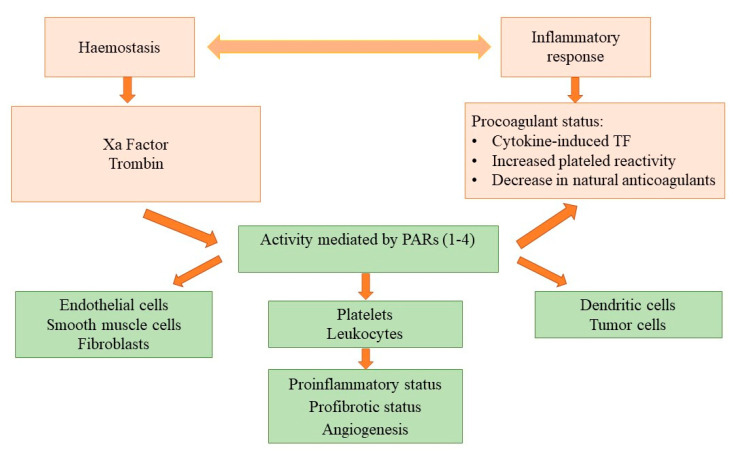
Schematic presentation of tumor angiogenesis (modified from others [36]). TF—tissue factor; PARs—protease-activated receptors.

The antiangiogenetic effect was the first to be targeted in experimental studies. Angiogenesis is a consequence of inflammation and hemostatic disturbances and plays an essential role in tumor progression. Inflammation has a prothrombotic effect as it decreases natural anticoagulants, activates platelets, or increases tissue factor (TF) expression. Moreover, both factor Xa (which represents the target of DOACs) and thrombin initiate and maintain angiogenesis and tissue fibrosis, thus promoting tumoral growth [36]. A schematic presentation of tumor angiogenesis is present in Figure 2. Therefore, experimental studies aimed at demonstrating the antitumoral growth effect of DOACs. 

The results of these studies on reducing the progression of tumors and metastases after DOACs were different and depended on the timing of DOACs and the type of cancer model used. Most of these experimental studies were performed with anticoagulant drugs that are only used in vitro [47]. We selected studies using DOACs approved for use in clinical practice.

The experimental in vitro and in vivo studies were extensively reviewed by Grandoni et al. [36]. Later, Najidh et al. identified nine publications that included a total of 19 in vivo experiments focusing on the effect of DOACs on tumor growth and metastasis [47]. These were animal models of fibrosarcoma, colorectal cancer, pancreatic cancer, melanoma, and breast cancer. Currently, dabigatran, rivaroxaban, and apixaban are the DOACs that have shown an angiogenetic effect.

Dabigatran acts by inhibiting thrombin, and studies on animals have demonstrated a decrease in tumor progression in breast cancer models when treatment is given immediately after the grafting of the neoplastic cells [47].

It has been proved that the timing of administering the treatment is an extremely important fact. However, two other experimental studies in breast cancer in animals showed that if dabigatran was administered when tumors were already established, it had no effect on the development of the primary tumor or on metastases. In a study of pancreatic cancer in mice, it was observed that if dabigatran was administered 1 week after inoculation of the cancer cells, it caused tumor growth, which is explained by the increase in intratumoral bleeding [47].

Rivaroxaban is included in the guidelines for the management of patients with cancer-associated VTE; however, studies showed different results depending on the cancer type. Graf et al. observed that rivaroxaban (0.4 mg/g chow diet) reduced fibrosarcoma weight by 50% and lung metastasis by 70% at 8 days after administration [62]. On the other hand, Maqsood et al. showed that rivaroxaban (0.5 mg/g chow diet) had no effect on tumor size or tumoral cell proliferation (quantified by Ki-67-positive tumoral cells) when injected in a xenograft model of pancreatic cancer cells [63].

Another study compared the effect of LMWH (dalteparin and tinzaparin) with DOACs (apixaban and rivaroxaban) on cell proliferation and tumor growth in vitro and in vivo in melanoma and pancreatic cancer models. Dalteparin and tinzaparin reduced tumor growth and tumor invasion in vitro. Apixaban and rivaroxaban treatment did not influence tumor development. A study using the chorioallantoic membrane assay model in vivo demonstrated that in vivo LMWH had a beneficial effect in terms of reducing tumor vascularity and apixaban reduced the rate of tumor proliferation and tumor growth. The results of these studies were explained by the fact that LMWH acts on tumor development and tumor vascularization by inhibiting factor Xa, inhibiting NFκB cell activity, and decreasing tumor and endothelial cell adhesion through selectins and ICAM receptors [48].

Additional studies are required to determine the potential benefit of DOACs for primary tumor or distant metastasis reduction in laboratory settings before performing clinical studies.

### 4.5. Some Practical Considerations on the Use of DOACs in Cancer Patients

#### 4.5.1. The Length of the Treatment

According to the current guidelines, the length of the anticoagulant treatment is 3–6 months. The treatment should be continued indefinitely in patients with active cancer, under chemotherapy, and multiple VTE risk factors [41,42,46]. The decision whether to extend the treatment should be reached following the periodic assessment of the hemorrhagic risk against the benefit of the treatment [45,46]. 

In cases where the VTE-recurrence risk is high, the anticoagulant treatment is administered over an extended period of time (over 3–6 months) [43,46,64]. Various risk scores may be used to stratify the patients’ recurrence risk vs. bleeding risk. The Ottawa score, for example, evaluates the 6-month VTE-recurrence risk considering the following variable: sex, cancer type, and previous history of VTE. In the modified score, female sex, lung cancer, and previous history of VTE is each given 1 point, breast cancer and cancer stage I and II receive −1 point [65]. 

The treatment for these patients needs to be structured according to the location of the neoplasm, major organ functions, patient compliance, concurrent medications, and the existence of other specific factors. For catheter-associated thrombosis, anticoagulant therapy should be administered if a catheter is in place. The recommended total duration of the therapy is at least 3 months [46]. For patients with VTE and cancer, extended anticoagulation (beyond the first 6 months) should be considered for an indefinite period or until the cancer is cured, and the therapeutic dose should be maintained [42,62].

#### 4.5.2. High Risk of Major Bleeding

The hemorrhagic risk factors that must be taken into consideration are an age 65>, prolonged periods of immobilization, the presence of metastases, renal impairment (creatinine clearance below 30 mL/min), and history of digestive hemorrhages, hemorrhagic diathesis, and thrombocytopenia [3]. Currently, no bleeding scores are implemented; however, the IMPROVE score is an example of a bleeding score that is useful in clinical [41] practice. It comprises the following: history of gastroduodenal ulcer, bleeding in the 3 months prior, admission platelets <50,000/mm^3^, liver failure, ICU/CCU stay, CV catheter, active cancer, or rheumatic disease [66]. 

Thrombocytopenia is frequent in neoplastic patients. Its presence increases the risk of bleeding but does not decrease the risk of developing thrombosis. The current guidelines recommend that the anticoagulant treatment be continued, without dose reduction, in cases in which the platelet count exceeds 50,000/mm^3^. DOACs can be administered at a platelet count >50,000/mm^3^, and when the platelet level drops under 50,000/mm^3^, LMWH should be introduced instead. Each decision should be individualized [67]. In accordance with the ISTH and NCCN guidelines, anticoagulation should be stopped when the platelet count is lower than 25,000/mm^3^, while the ASCO guidelines recommend the withdrawal of the anticoagulants at a threshold of 20,000/mm^3^ [41,44,46].

According to the current guidelines, DOACs are to be avoided in patients with a high risk of bleeding (luminal gastrointestinal, genitourinary, hematological neoplasms, with nephrostomy, or with an active gastroduodenal ulcer), and LMWHs should be administered instead [41,42].

#### 4.5.3. Renal Impairment

The administration of DOACs is not generally recommended in the presence of renal impairment with a <15 mL/min/m^2^ creatinine clearance. Under careful monitoring, in patients with a creatinine clearance of 15–50 mL/min/m^2^, the following may be used: apixaban 2 × 2.5 mg/day, edoxaban (30 mg/day), and rivaroxaban (15 mg/day).

Although there are attempts to use DOACs in dialysis patients (apixaban 2 × 2.5 mg), the use of DOACs in the case of a creatinine clearance below 15 mL/min/m^2^ and dialysis is contraindicated [45,59]. For apixaban, a dose reduction is recommended when two of the following three criteria are met: age ≥ 80 years, weight ≤ 60 kg, and serum creatinine ≥ 1.5 mg/dL [41].

There are certain issues regarding DOACS dosing in RCT for VTE. Patients with CrCl < 25 mL/min were excluded from the trials testing apixaban, whereas patients with CrCl < 30 mL/min were excluded from those investigating rivaroxaban, edoxaban, and dabigatran. The dosages of dabigatran, rivaroxaban, and apixaban were not reduced in patients with mild–moderate renal dysfunction (CrCl between 30–60 mL/min), whereas edoxaban was given at a 30 mg dose in these patients [42]. During the first 3 weeks of treatment, it is not recommended to adjust the dose of Rivaroxaban, it should be maintained at 2 × 15 mg/day, or it should be replaced by LWMH in cases with severe renal impairment.

Grandone et al. have recently published a statement paper regarding the use of DOACs in patients with renal impairment. The paper elegantly summarized the recommendation of three different international societies (2018–2019) regarding the dosing of DOACs adjusted to creatine clearance and reviewed the real-life clinical trials focusing on DOACs in patients with renal impairment. The authors concluded that DOACs are recommended in patients with CKD, but close monitoring is necessary in order to avoid CRNMB [68].

#### 4.5.4. Liver and Gastrointestinal Diseases

The oral intake of DOACs is an important drawback, as the absorption might be impaired by nausea and vomiting, symptoms which are relatively frequent in neoplastic patients undergoing chemotherapy.

DOACs absorption is influenced by the anatomic changes in the digestive tube. Rivaroxaban, dabigatran, and edoxaban must be avoided in patients with gastrectomies because they are absorbed in the distal part of the stomach and the proximal part of the small intestine, and they are dependent on gastric acidity for absorption. In such instances, apixaban should be administered because it is absorbed in the distal small bowel and in the ascending colon and is not pH-dependent for absorption [61].

Apixaban should be avoided in patients with intestinal resections and colectomies. Rivaroxaban, dabigatran, or edoxaban should be administered instead. Dabigatran is administered as capsules and should be avoided in the case of tube-fed patients [61].

Apixaban is not to be administered in cases in which transaminase levels are elevated to more than twice the upper limit, while rivaroxaban and edoxaban are not recommended when transaminase levels are three times the upper limit [61].

#### 4.5.5. Interaction with Other Drugs

DOACs have fewer interactions than antivitamin K medication.

Drugs that induce CYP3A4, a cytochrome P450 component present in hepatic cells (rifampicin, carbamazepine, phenobarbital, valproic acid, doxorubicin, vinblastine, sunitinib, vandetanib, and dexamethasone) decrease the anticoagulant effect of apixaban and rivaroxaban. The drugs that inhibit CYP3A4 (dronedarone, diltiazem, clarithromycin, erythromycin, crizotinib, cyclosporine, and tacrolimus) increase the anticoagulant effect of rivaroxaban and apixaban [46].

The P glycoprotein (P-gp) transporter influences and modifies the absorption of dabigatran in the intestines. The P-gp inductors decrease the absorption and the anticoagulant action of dabigatran (rifampicin, dexamethasone, carbamazepine, phenobarbital, phenytoin, levetiracetam, valproic acid, doxorubicin, vinblastine, sunitinib, and vandetanib). The P-gp inhibitors (dronedarone, verapamil, amiodarone, quinidine, clarithromycin, erythromycin, ticagrelor, tacrolimus, cyclosporine, imatinib, and crizotinib) increase dabigatran absorption and its anticoagulant action. The edoxaban dose should be lowered to 30 mg/day when the P-gp inhibitors are concomitantly administered [69]. The interaction of DOACs with antineoplastic therapy is not fully understood and has not yet been investigated in large clinical trials. Bellesoeur et al. analyzed this interaction using the scarce evidence generated by real-life trials. They concluded that “the risk of pharmacokinetic drug–drug interaction with DOAC should be estimated taking account for clinical and biological parameters such as age, sarcopenia, inflammation, renal and hepatic impairment” [70].

#### 4.5.6. Extreme Weight

In special cases such as extreme weight, which is frequent in the presence of neoplasia, pharmacological interferences, or suspected noncompliance, monitoring of the DOAC activity may be required. The recommended test for monitoring xabans is the chromogenic antifactory Xa assay. The ranges are 12–137 ng/mL for rivaroxaban and 34–230 ng/mL for apixaban. The recommended tests for monitoring dabigatran are the dilute thrombin time (DTT) test and the ecarin clotting time (ECT) test [46]. For patients weighing less than 60 kg, a lower dose of edoxaban (30 mg/dl) should be administered [42].

## 5. Conclusions

The anticoagulant treatment of cancer-associated VTE is difficult to administer because patients have a high risk of recurrence and bleeding.

DOACs exhibit a similar efficiency as VKAs for the treatment of cancer-associated VTE and a lower risk of bleeding. DOACs are more efficient and easier to administer as compared to LMWHs in the prevention of VTE recurrence, but they can be associated with a higher risk of bleeding.

Thus, we support the administration of DOACs as the first-choice treatment in cancer-associated VTE in patients who have a low risk of bleeding under anticoagulant treatment, who do not have severe renal impairment, and who are not undergoing treatments that could interact with the DOAC mechanism of action.

DOACs may also have an antineoplastic effect which depends on the type of cancer and on the early start of the treatment, but more studies are required to support this hypothesis.

## Figures and Tables

**Table 1:** Meta-analysis published in the past 6 years.

Meta-Analysis	Publication Year	Results
1. Sardar, P. [7]	2015; 19,832 pts and 1197 pts.	DOACs administered in patients with cancer were found to be as safe and efficient as is the case in patients without cancer. Rivaroxaban might be effective and safe in patients with cancer, as compared to VKA [6].
2. Posch, F. [8]	2015; 3242 pts	DOACs have similar efficacity and safety to LMWH in patients with cancer [7].
3. Mantha, S. [9]	2015	Apixaban appears to be safer than other oral anticoagulants, with a lower risk of bleeding in patients with cancer [8].
4. Boonyawat, K. [10]	2017; 98.244 pts	When the influence of body weight on DOAC efficiency was assessed, the results showed that it is not recommended to adjust the doses of DOACs outside the already known limits [9].
5. Kahale, LA. [11]	2017; 1486 pts	DOACs do not decrease mortality in cancer patients but may be responsible for more bleeding events [10].
6. Kahale, LA. [12]	2018; 5167	For long-term therapy for cancer-associated thrombotic events, DOACs, as compared to LMWH, may have an efficient antithrombotic effect, but safety issues arise because of an increased risk of major bleeding [11].
7. Vedovati, MC. [13]	2018; 1430 pts	In patients with cancer and VTE, DOACs were showed to be safe and efficient as compared to LMWHs [12].
8. Xing, J. [14]	2018; 667 pts	Rivaroxaban proved to be efficient and safe as compared to LWMH (enoxaparin for the prevention of recurrent thrombotic events in neoplastic patients). Thus, rivaroxaban was recommended as a therapeutic option for cancer-associated VTE [13].
9. Martinez, BK. [15]	2018; 949 pts	Rivaroxaban demonstrated similar levels of safety (rate of major bleeding) and efficiency (recurrent VTE) to the other anticoagulants for patients with cancer-associated VTE. The mortality was lower than reported in many anticoagulation CAT trials [14].
10. Park, H. [16]	2018	For the treatment of cancer-associated VTE, DOACs proved to be safer and more efficient as compared to VKA. DOACs could be one of the standard therapeutic options in neoplastic patients. Among DOACs, apixaban exhibited a better outcome [15].
11. Hong, Y. [17]	2018	Rivaroxaban was associated with a lower hospital admission rate as compared to LWMH [16].
12. Gómez-Outes, A. [18]	2018; 29,844 pts	The type of anticoagulation made no difference in the overall survival or causes of death, while the presence of active cancer was associated with a poor outcome and a higher mortality rate [17].
13. Li, A. [19]	2019	DOACs proved to be more effective than LMWHs in secondary prevention of VTE. Unfortunately, DOACs had a low safety profile as a result of increasing the risk of major bleeding and CRNMB, even though the absolute risk differences were small (2–3%). Better compliance with DOACs than LMWHs was hypothesized to explain the differences in bleeding events [18].
14. Rossel, A. [20]	2019; 4667 pts	DOACs proved efficient in secondary prevention of VTE in neoplastic patients but showed a low safety profile with an increased risk of bleeding as compared to LMWH [19].
15. Kirkilesis, GI. [21]	2019; 6980 pts	DOACs were more effective than LMWHs in preventing VTE recurrence but may carry a higher risk of major bleeding [20].
16. Massimiliano, Camilli [22]	2020, 2894 pts	As compared to LMWH, DOACs were associated with a significantly lower risk of VTE recurrence and were not associated with an increased risk of major bleeding; however, they were associated with an increased risk of nonmajor bleeding and gastrointestinal bleeding [21].
17. Desai, R. [23]	2020, 18,945 pts	DOACs proved to be more effective in secondary prevention of VTE and were associated with a small risk of CRNMB. DOACs were considered to only be safe in the appropriately selected neoplastic patients [22].
19. Sabatino, I. [24]	2020; 2907 pts	DOACs demonstrated similar efficiency and safety to dalteparin in preventing CAT VTE recurrence. However, DOACs were associated with higher rates of nonmajor bleeding as compared with dalteparin, primarily in patients with gastrointestinal malignancies [23].
20. Desai, A. [25]	2020; 4341 pts	DOACs were efficient in terms of lowering the risk of VTE or recurrent VTE in patients with cancer but demonstrated safety issues regarding the increased risk in major and nonmajor bleeding events without influencing the survival rate [24].
21. Molik, F. [26]	2020; 2894 pts	DOACs for VTE treatment and secondary prophylaxis in neoplastic patients proved to be more effective than LMWH but with safety issues related to major and nonmajor bleeding events, especially in those with digestive cancers [25].

CAT—cancer-associated thrombosis; CRNMB—clinically relevant nonmajor bleeding; DOAC—direct oral anticoagulant; PE—pulmonary embolism; VKA—vitamin K antagonist; LMWH—low-molecular-weight heparin; VTE—venous thromboembolism.

**Table 2:** Randomized controlled studies comparing DOACs to VKAs and LMWH, respectively.

Name of Study	DOAC	Active Cancer Randomization (*n*)	Efficacy End Point (Recurrent VTE) Rate HR (95% CI)	Safety End Point (Major Bleeding) Rate HR (95% CI)
**DOAC vs. VKA**				
1. RE-COVER I/II [27]	Dabigatran	114 vs. 107	3.5% vs. 4.7% 0.74 (0.20–2.7)	13% vs. 9% 1.48 (0.64–3.4)
2. EINSTEIN-DVT/PE [28]	Rivaroxaban	258 vs. 204	2% vs. 4% 0.62 (0.21–1.79)	12% vs. 13% 0.82 (0.48–1.38)
3. AMPLIFY [29]	Apixaban	88 vs. 81	3.7% vs. 6.4% 0.56 (0.13–2.37)	2.3% vs. 5% 0.45 (0.08–2.46)
4. HOKUSAI-VTE [30]	Edoxaban	85 vs. 77	2% vs. 9% 0.30 (0.06–1.51)	19% vs. 26% 0.66 (0.34–1.27)
DOAC vs. LMWH				
5. SELECT-D [31]	Rivaroxaban	203 vs. 203	4% vs. 11% 0.43 (0.19–0.99)	6% vs. 4% 1.83 (0.68–4.96)
6. XALIA [32]	Rivaroxaban	146 vs. 223	3.4% vs. 4.5%	1.4% vs. 3.6%
7. MSK [33]	Rivaroxaban	200	4.4%	2.2%
8. ADAM-VTE [34]	Apixaban	145 vs. 142	3.4% vs. 14.1% 0.26 (0.09–0.80)	0% vs. 2.1% *p* = 0.9956
9. HOKUSAI-VTE CANCER [30]	Edoxaban	522 vs. 524	7.9 % vs. 11.3% *p* = 0.09	6.9% vs. 4% *p* = 0.04
10. CARAVAGGIO [35]	Apixaban	576 vs. 579	5.6% vs. 7.9% 0.63 (0.37–1.07)	3.8% vs. 4.0% 0.82 (0.40–1.69)
11. CANVAS [36]	Rivaroxaban, Apixaban, Edoxaban, Dabigatran	811	ongoing study	ongoing study
12. CONKO-011 [37]	Rivaroxaban	450	ongoing study	ongoing study
13. CASTA-DIVA [38]	Rivaroxaban	159	ongoing study	ongoing study
14. COSIMO [39]	Rivaroxaban	528	ongoing study	ongoing study

CI—confidence interval; DOAC—direct oral anticoagulant; HR—hazard ratio; PE—pulmonary embolism; VKA—vitamin K antagonist; LMWH—low-molecular-weight heparin; VTE—venous thromboembolism.

**Table 3:** Guidelines published in the past 6 years.

Society	Recommendations
1. Treatment algorithm in cancer-associated thrombosis: Canadian expert consensus (2018) [40]	1. Does not mention anticoagulation counterindications. 2. DOACs are preferred to LMWH if the hemorrhagic risk is low, in the absence of gastrointestinal tumors, genitourinary tumors, and if there are no drug interactions. 3. The treatment is recommended for 3 months with re-evaluation at the end of the treatment for cancer evolution and type (active/inactive).
2. ISTH (2018) [41]	1. We suggest the use of specific DOACs (edoxaban or rivaroxaban) for acute VTE in patients with cancer who present a low risk of bleeding and no drug–drug interactions with current systemic therapy. LMWHs are considered an acceptable alternative. 2. Currently, edoxaban and rivaroxaban are the only DOACs with RCT evidence when compared to LMWH in cancer populations. 3. We suggest the use of LMWHs in cancer patients with an acute diagnosis of VTE and high risk of bleeding (luminal gastrointestinal cancer, genitourinary tract cancer, bladder or nephrostomy tubes, or in patients with active gastrointestinal mucosal abnormalities, such as duodenal ulcers, gastritis, esophagitis, or colitis). 4. We recommended individualized treatment by including patients’ preferences and values.
3. ESC (2019) [42]	1. Weight-adjusted subcutaneous LMWH should be considered for the first 6 months over VKAs (IIa A). 2. Edoxaban should be considered as an alternative to LWMH in patients without gastrointestinal cancer (IIa B). 3. Rivaroxaban should be considered as an alternative to LWMH in patients without gastrointestinal cancer (IIa C). 4. Extended anticoagulation (> 6 months) should be considered for an indefinite period or until cancer is cured (IIa B). 5. Incidental PE should be managed as symptomatic PE if it involves segmental or more proximal branches, multiple subsegmental vessels, or a subsegmental vessel in association with confirmed DVT.
4. ACCP (2019) [43]	1. We suggest the use of specific DOACs for cancer patients with an acute diagnosis of VTE, low risk of bleeding, and no drug–drug interactions with current systemic therapy. LMWHs are an acceptable alternative. 2. Currently, edoxaban and rivaroxaban are the only DOACs with RCT evidence when compared to LMWH in cancer populations. 3. We suggest the use of LMWHs for cancer patients with an acute diagnosis of VTE and a high risk of bleeding (luminal gastrointestinal cancer and genitourinary tract cancer).
5. ASCO (2019) [44]	1. Initial anticoagulation may involve LMWH, UFH, fondaparinux, or rivaroxaban. LMWH is preferred over UFH for the initial 5–10 days of anticoagulation (evidence quality: high; strength of recommendation: strong) in patients initiating treatment with parenteral anticoagulation. 2. For long-term anticoagulation treatment, LMWH, edoxaban, or rivaroxaban are preferred for at least 6 months because of improved efficacy over VKAs (evidence quality: high; strength of recommendation: strong). 3. Anticoagulation with LMWH, DOACs, or VKAs beyond the initial 6 months should be offered to patients with active cancer, such as those with metastatic disease or those receiving chemotherapy. 4. The insertion of a vena cava filter may be offered as an adjunct to anticoagulation in patients with progression of thrombosis despite optimal anticoagulant therapy. 5. Incidental PE and deep vein thrombosis should be treated in the same manner as symptomatic VTE, given their similar clinical outcomes when compared to cancer patients with symptomatic events. 6. Anticoagulant use is not recommended in order to improve survival in patients with cancer without VTE.
6. Thrombosis and Hemostasis Society of Australia and New Zealand (2019) [45]	1. For DVT or PE that is provoked by active cancer, treatment with therapeutic LMWH for at least 6 months should be administered (evidence: high; strength of recommendation: strong). 2. Patients with incidental PE should be treated in a similar way to patients with symptomatic cancer-associated thrombosis. 3. Edoxaban and rivaroxaban have been shown to be as efficacious as dalteparin in cancer-related thrombosis, but they are associated with an increased risk of major bleeding or CRNMB and, therefore, can be considered when appropriate.
7. NCCN Guidelines Insights Cancer-Associated Venous Thromboembolic Disease (2019) [46]	1. For noncatheter-associated DVT or PE, indefinite anticoagulation should be recommended while cancer is active, under treatment, or if risk factors for recurrence persist. 2. Apixaban is an option for anticoagulation in patients with cancer and should be limited to patients who refuse or have compelling reasons to avoid LMWH. 3. LMWH/UFH plus dabigatran is a potential treatment option for cancer-associated VTE and should be limited to those patients who refuse or have compelling reasons to avoid long-term LMWH. 4. LMWH followed by edoxaban is the first option for anticoagulation in cancer-associated VTE. 5. Rivaroxaban is an option for anticoagulation treatment of VTE in patients with cancer. Unlike single-agent apixaban, it is not limited to patients with compelling reasons to avoid LMWH. 6. For catheter-associated thrombosis, anticoagulant therapy should be administered if a catheter is in place. The recommended total duration of the therapy is at least 3 months.

ISTH—International Society of Thrombosis and Hemostasis; ESC—European Society of Cardiology; ACCP—American College of Clinical Pharmacy; ASCO—American Society of Clinical Oncology; NCCN—National Comprehensive Cancer Network; DOAC—direct oral anticoagulant; VTE—venous thrombo-embolism; LMWH—low-molecular-weight heparins; UFH—unfractionated heparin; CNRMB—clinically relevant nonmajor bleeding; PE—pulmonary embolism; VKAs—vitamin K antagonists; RCT—randomized controlled trials.

**Table 4:** The studies that showed a favorable antineoplastic effect of DOACs in experimental model.

Authors	Cancer Model/No. of Animals (Mice) Per Experimental Group	Treatment (Dose, Mode, Duration, and Timing)	Results
DeFeo et al. (2010) [47]	syngeneic; orthotopic breast cancer model/4–10	Dabigatran [45 mg/kg body weight twice a day (Mon–Fri) or 60 mg/kg once a day (Sat, Sun) by oral gavage for 4 weeks, beginning 1 day before tumor cell injection]	Reduced liver micrometastases (no significant effect on lung micrometastases)
Graf et al. (2019) [47]	syngeneic; s.c. fibrosarcoma model/9	Rivaroxaban [0.4 mg/g chow diet for 8 days, started 14 days after cancer cell inoculation]	±50% reduction in tumor weight ±70% reduction in no. of macroscopic lung metastases
	syngeneic; s.c. colorectal cancer model/9–11	Rivaroxaban [0.4 mg/g chow diet for 9 days, started 12 days after cancer cell inoculation]	±40% reduction in tumor volume
	spontaneous; breast cancer model/28	Rivaroxaban [0.4 mg/g chow diet for 7 weeks, started from week 13 after birth]	reduction in no. of lung metastases
Sophie Featherby (2019) [48]	syngeneic the chorioallantoic membrane (CAM) model/3	Apixaban (1 µg/mL) Rivaroxaban (0.6 µg/mL)	Apixaban (1 µg/mL) partially reduced the growth of the implanted tumors

no.—number.

## Data Availability

Not applicable.
